# 1404. Remotely Piloted Aircraft & Quadcopters used in Combating Malaria/Dengue in desert terrain

**DOI:** 10.1093/ofid/ofad500.1241

**Published:** 2023-11-27

**Authors:** V S Srikanth

**Affiliations:** 15AFH, jaisalmer, Rajasthan, India

## Abstract

**Background:**

Malaria is a major vector borne disease across the world. The major problem we are facing is difficulty in elimination of source. WHO World Malaria Day theme is ***“Harness innovation to reduce the malaria disease burden and save lives”*** which is the only way to eradicate malaria by using modern technologies. We used remotely piloted aircraft and quadcopters for effective malaria surveillances and also created a task force **DMSEU** (Drone Malaria Surveillance and eradication unit). This is first of its kind study where multimode surveillances was done using different types of UAV.

**Methods:**

This is an observation study during malaria outbreak during Oct 2022 – Dec 2022. DMSEU was formed. Mosquito breeding locations were identified based on map marking of the areas from where clusters of malaria cases were reported. Then satellite images of locations were evaluated for gross land scape assessment. Based on this assessment RPAs were used for identification of breeding sites with the help of the the imagery systems like FLIR and Day CAM. In our unit Quad copters were used for assessment of breeding sites.

**Results:**

Ramgarh was identified as an area of source of malarial outbreak based on cluster mapping. When the satellite images of Ramgarh was analysed, it showed a dense vegetation developed near TV tower area during rains. RPA was flown over that area to find out exact breeding sites using the coordinates. We also noticed that there were many drip irrigation fields. When RPA were flown over the area, it was identified that each field had an open water storage tank for drip irrigation. Using Quad copter, we did further assessment at each roof tops for potential breeding sites like open overhead tank, water stagnation

RPA - Surveillance of malaria breeding site

**Figure:**
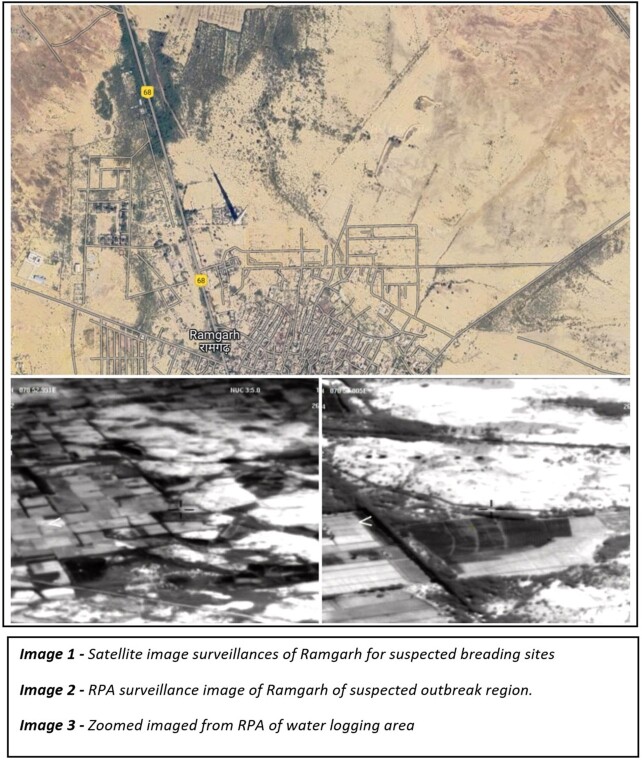

Quardcopter Malaria Breeding site surveillance

**Figure:**
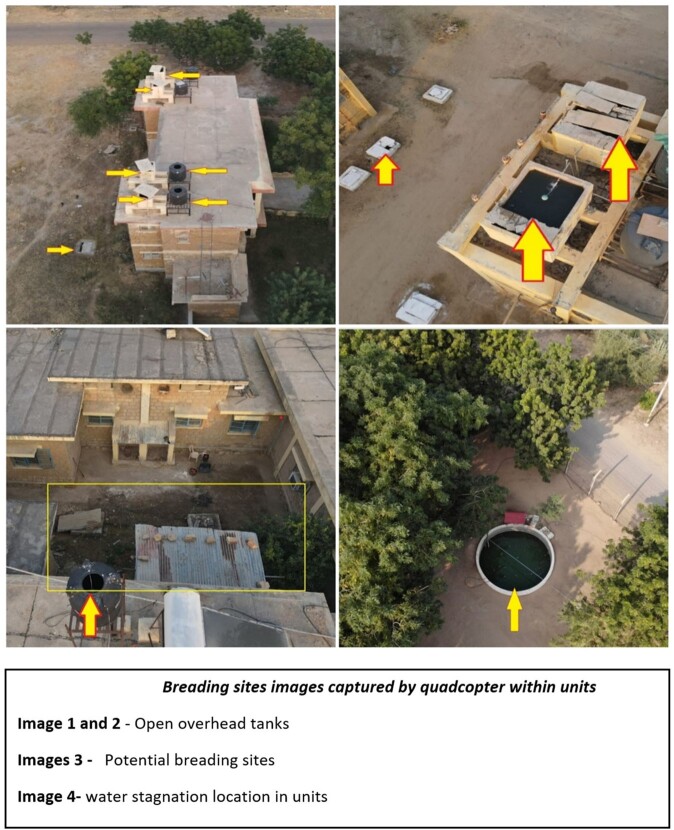

**Conclusion:**

The malaria menace can be brought to an end only by using newer technologies and multi-sector collaboration working hand in hand. Using different types of UAV like RPA and Quadcopter was very effective in source identification and eradication. To improve the scope of area coverage and efficiency of mosquito breeding sites eradication, new drone should be built with upgraded capability of spraying and fogging capacities. Using quadcopters and drone played a major role in eliminating them and curtailing the cases drastically in a very short span of time in effective way and also saved manpower and time.

**Disclosures:**

**All Authors**: No reported disclosures

